# Investigating Public trust in Expert Knowledge: Narrative, Ethics, and Engagement

**DOI:** 10.1007/s11673-016-9767-4

**Published:** 2017-01-31

**Authors:** Silvia Camporesi, Maria Vaccarella, Mark Davis

**Affiliations:** 1 Bioethics and Society, Department of Global Health and Social Medicine, King’s College London, WC2R 2LS London, UK; 2 Medical Humanities, Department of English, University of Bristol, 3-5 Woodland Road, Clifton, Bristol, UK; 3 School of Social Sciences, Monash University, Wellington Road, Clayton, VIC 3800 Australia

**Keywords:** Public trust, Trust, Expertise, Expert knowledge, Narrative, Biopolitics

## Abstract

“Public Trust in Expert Knowledge: Narrative, Ethics, and Engagement” examines the social, cultural, and ethical ramifications of changing public trust in the expert biomedical knowledge systems of emergent and complex global societies. This symposium was conceived as an interdisciplinary project, drawing on bioethics, the social sciences, and the medical humanities. We settled on public trust as a topic for our work together because its problematization cuts across our fields and substantive research interests. For us, trust is simultaneously a matter of ethics, social relations, and the cultural organization of meaning. We share a commitment to narrative inquiry across our fields of expertise in the bioethics of transformative health technologies, public communications on health threats, and narrative medicine. The contributions to this symposium have applied, in different ways and with different effects, this interdisciplinary mode of inquiry, supplying new reflections on public trust, expertise, and biomedical knowledge.

## Introduction: Why Public Trust and Expert Knowledge, Now?

“Public Trust in Expert Knowledge: Narrative, Ethics, and Engagement” examines the social, cultural, and ethical ramifications of changing public trust in the expert biomedical knowledge systems of emergent and complex global societies. This symposium was conceived as an interdisciplinary project, drawing on bioethics, the social sciences, and the medical humanities. For us, trust is simultaneously a matter of ethics, social relations, and the cultural organization of meaning. The contributions to this symposium have applied, in different ways and with different effects, this interdisciplinary mode of inquiry, supplying new reflections on public trust, expertise, and biomedical knowledge.

The idea for this symposium emerged during a doctoral course sponsored by a King’s College London (KCL) initiative for innovative interdisciplinary training across the humanities and social sciences (KISS-DTC) run by Camporesi and Vaccarella at KCL in 2015 and attended by Davis on a visiting fellowship. We discovered a shared commitment to narrative inquiry across our fields of expertise in the bioethics of transformative health technologies, public communications on health threats, and narrative medicine. In 2016, an Interdisciplinary Research Award from the Faculty of Arts, Monash University allowed us to deepen our collaboration in the form of workshops on narrative inquiry held at Monash University (Melbourne, Australia) and this symposium with the *Journal of Bioethical Inquiry*.

We settled on public trust as a topic for our work together because its problematization cuts across our fields and substantive research interests. Trust in expert biomedical knowledge (i.e. willingness to believe, endorse, and enact expert advice) has emerged as an ethical, epistemological, and political problem for governments seeking to engage and influence publics on matters as wide-ranging as public policy on the environment and economic development, biopolitics, and well-being over the life course. It is apparent that there is considerable flux in the trust invested in health experts, in their formation, and in the emerging technologies and science on which their authority is, in part, based.

For example, great emphasis is currently being placed on the trustworthiness of the scientists and regulators who are pioneering CRISPR, a new genome editing technology that has the potential to alter the genomic character of all life (Doudna and Charpentier 2014). Disease outbreaks like Zika virus in 2015–2016 are in part constituted in media reports on the rapidly changing epidemiology, microbiology, and clinical management of the disease, where questions of uncertainty and therefore trust in expert knowledge are foregrounded (Briggs and Hallin 2016). The efforts to manage the Zika pandemic link back to genome editing: CRISPR, for example, is conceptualized as one means of eradicating mosquito species which transmit the virus (Brooks 2016). This way of applying new scientific knowledge to health threats is infused with questions of trust, particularly in the face of the uncertainties implied in gene editing technologies, the vagaries of virus outbreaks, like Zika, and of the long-term consequences of intervening in the biosphere with genome editing technologies. More generally, how this story of technological possibility and uncertainty is narrated, in media outlets and in cultural products, also impacts public perception and trust in biomedical expertise and knowledge. By drawing on the educational potential of these textual sources for healthcare professionals, narrative medicine, in part, aims to consolidate trust in the beneficence which underpins healthcare, and in the expert-lay relationship which is fundamental to care in medicine. If contemporary biomedical practices are increasingly technologized, instrumentalized, and bureaucratized, fictional accounts of new technologies and the (auto)pathographies of patients and carers explore, challenge, and reinvent these expert–lay relationships of trust and care.

In what follows we take up a critical stance on trust in connection with some reflections on the contributions to this symposium. We have selected five papers for our interdisciplinary, international symposium: from Canada, Singapore, and the United States, Daniel Buchman, Anita Ho, and Daniel Goldberg address the nexus of phenomenology, trust, and emerging pain management technologies. From Australia, Katie Atwell, Julie Leask, Samantha Myer, Philippa Rokkas, and Paul Ward discuss their sociological research on the resistance to vaccination technologies articulated with distrust of the pharmaceutical industry. Also from Australia, Karen-Anne Wong’s paper presents a Science, Technology, and Society perspective on the circulation and commodification of gametes in new modes of reproductive health, race, and gender. From the United States, Jennifer Edwell and Jordynn Jack examine women’s narratives on gestational diabetes, maternity care, expert advice, and their implied identities as untrustworthy mothers. From England, Deborah Bowman—a public bioethicist and medical humanities scholar—reflects in her article on innovative modes of deliberative public engagement with science, ethics, and trust materialized in theatre and radio. The papers share a commitment to 1) the articulation of the meanings and related social and ethical implications of trust in specific settings, 2) reflection on the role of narrative in the construction of public trust, and 3) examination of the ways in which publics engage (or not) with expert knowledge. In what follows, we reflect on these three themes and make links with the contributions, helping the reader to explore the synergies and nuances of this symposium.

## Trust, Its Meanings and Implications

Trust pervades personal, social, and political life and has many meanings and effects. Basic trust is seen in some framings as the foundation of self and ontological security—that is, trust as a sense of being in the world garnered during early life and in primary relationships with nurturing others (Giddens 1990, 1991). Trust figures in the everyday reciprocity of social relations, for example, in economic systems where labour is exchanged for a wage and in the everyday disclosures and confidences of conversation and relationships. As previous comments implied, governance and politics are imbued with questions of trust and distrust (Luxon 2015), as are public engagements with the institutions which broker scientific expertise, such as the National Health Service in the United Kingdom (Calnan and Rowe 2008). The expertise and authority of science is also an object of trust, since so many of the life choices individuals are required to make depend on the knowledge possessed by others (Giddens 1990, 1991).

Across the social sciences, humanities, and bioethics, trust is sometimes the object of inquiry, but at other times it is a taken for granted concept with the status of terms like “society” or “community.” For example, a Google Scholar search on trust shows it to be a much used term (2.9 million hits and counting as of October 2016), the meaning of which appears to dissipate through its proliferation. We endeavour to trouble the taken-for-granted status of trust by foregrounding and nuancing it through the critical lenses of the contributions for this symposium.

Trust is also much psychologized and quantified. For example, a United States survey on public responses to the 2009 H1N1 pandemic (Freimuth et al. 2013) operationalized trust as an aggregate of measures of the extent to which the government and its spokespersons were regarded as “committed,” “caring and concerned,” “open,” “competent,” “honest,” “interested in citizens’ best interest,” and “would act to protect citizens.” The survey researchers found that respondents who exhibited lower trust were less likely to comply with public health advice. This idea that trust varies along a continuum implies “hydraulic” conceptualizations of society, such as in notions of high and low trust societies (Fukuyama 1995) and similar ideas that, for example, social capital is more or less present in particular forms of collective organization (Putnam 2000), or the idea that individuals and communities are more or less resilient in the face of threats to their health and wellbeing (Sherrieb, Norris, and Galea 2010). This symposium challenges whether hydraulic notions of trust (low/high, continuums and gradients, absent/present) helpfully explain the complexities of trust in expert knowledge systems.

In addition, political theorist Russell Hardin has written that thinking of trust as more or less absent is nostalgic and utopian (Hardin 2006). The idea that collectives and societies have ever experienced absolute trust is likely to be mistaken and, relatedly, efforts to restore trust work towards an ideal that may not have ever been possible. For Hardin, politics are in a somewhat ironic and historically embedded relation with trust. In this view, politics are always post trust. By implication, it may be mistaken to assume that in the realm of biopolitics, trust can be retrieved and shaped towards an absolute ideal in an effort to protect and enhance the health of individuals and populations.

In bioethics, “trust” is referred to as one of the “founding pillars” of clinical research ethics. Edmund Pellegrino, Georgetown physician, bioethicist, and medical humanities pioneer, listed trust as one of the key and inalienable virtues of the good doctors in his virtue-based approach to bioethics (Pellegrino 1985, 1995). In a 1996 paper by Nancy Kass and colleagues (1996, 25), trust was defined as the “fragile foundation of contemporary biomedical research” and implicated in the horrific scandals of the Tuskegee Syphilis Study in the United States, as discussed by Beecher (1976). This notorious study—for which researchers exploited the trust usually ascribed to their profession and knowingly withheld diagnostic details and treatment from about 600 African American rural workers infected with syphilis—prompted the establishment of the U.S. National Committee for the Protection of Human Subjects and the consequent 1979 Belmont Report, which articulated three pillars of human subject research (respect for persons, beneficence, and justice).

Yet, as in the social sciences, trust is not all that it seems in bioethical perspective. Kantian philosopher and bioethicist Onora O’Neill, in her 2002 BBC Reith Lectures, dismantled three contemporary claims related to trust, namely: 1) that there has been a great decline in trust, 2) that we should have more trust, and 3) that we should rebuild trust. Echoing Hardin, O’Neill argued that the relation of trustworthiness, and therefore not trust as a state of being, should be seen as the basis of accountability and responsibility in public life. O’Neill provocatively noted that if the aim of society was to have “more trust,” that would be “a stupid aim” (O’Neill 2002), as trust can be misdirected and invested in unhelpful ways. Hence, she argued, what is important is not to increase the net amount of trust in our society but to secure and advance trustworthiness—that is, experts, institutions, and knowledge worthy of trust. This perspective shifts the gaze away from whether or not publics trust experts, science, and knowledge, towards the qualities and conduct of experts, the practices of science, and to knowledge itself. She added:

I would aim to have more trust in the trustworthy, but not in the untrustworthy. That means that what matters in the first place is not trust per se but trustworthiness, or whether something or someone is worth trusting in the first place. It is judging how trustworthy people are in particular ways. (O’Neill 2002)

Importantly for this symposium, are narrative figures in this relational politics of trustworthiness. Since the early days of narrative medicine, trustworthiness has been understood as one of the key areas of narrative intervention within medical education. As early as 2001, Rita Charon, leading U.S. scholar of narrative medicine, claimed that

… a scientifically competent medicine alone cannot help a patient grapple with the loss of health or find meaning in suffering. Along with scientific ability, physicians need the ability to listen to the narratives of the patient, grasp and honour their meanings, and be moved on the patient’s behalf. […] narrative competence […] enables the physician to practice medicine with empathy, reflection, professionalism and trustworthiness. Such a medicine can be called narrative medicine. (Charon 2001)

Essential to an engaged and empathic caring process, trustworthiness is said to originate from individual doctors’ narrative competence and to lead to a broader beneficial impact on medicine at large. But as with some social science and bioethical conceptualizations of trust, there is a utopian and absolutist quality to the perspective on trust afforded by narrative medicine and an implied romanticization of pathographers and their addressees. Insisting on the virtues of narrative in the clinic may paradoxically reinforce medical authority over the patient through the imperative to initiate narrative and especially a particular kind of disclosure leading to “healthy” self-transformation. Trust, in this framing of narrative medicine, becomes disciplinary. Following Foucault, American philosopher-physician Jeffrey Bishop emphasized that “Power is written into the very fabric of our relationships to ourselves, to others, and to social institutions. […] Medical humanism, like all other humanisms, promises intimacy, but is really about control” (2008, 21). Building upon Bishop’s critique, we note how much narrative medicine not so secretly relies on an instrumental view of humanistic knowledge, and trust can be controversially crucial in this instrumentalization: in its crudest form, the reasoning would go along the lines of “if by listening carefully to my patient’s story, I can persuade them to trust me, my patient will then do what I want.” It would, indeed, be problematic if trust was manoeuvred to impose biomedical priorities and mislead “difficult” patients, whom healthcare professionals and social workers might wish to control and discipline.

Each contribution to this symposium approaches trust from a critical perspective, as we do, and therefore offer the reader a range of tools for inquiry and insights. For Buchman and co-authors (2017), trust is axiomatic to the organization of the relationship between people with chronic pain and healthcare providers, but these patients and experts—epistemologically, experientially and agentially—are not equal in the production of approaches to the management of chronic pain. Chronic mistrust of the chronic pain sufferer—or notions of malingering—characterize the relationships between patients and expert providers. Buchman and colleagues make the point that this approach to chronic pain depends on a hierarchy of expert knowledge set above the feelings of the patient. Through “objectification” of the inherently and inescapably subjective experience of pain, the experiential expertise of the pain sufferer is marginalized and disrespected. The science of pain management, on which medical experts build their authority and power, is therefore a site for symbolic and material violence.

Parents who refuse childhood vaccination also sit in opposition to expert public health guidance, as Attwell and co-authors (2017) point out. Drawing on Giddens’s concept of trust (1990) and its implications for reflexive modernization, Attwell et al. question binary constructions of trust and distrust. For them, in their consideration of the experience narratives of vaccine refusers, trust is fundamentally relational and understood as a “web,” where trust or distrust in one system (the pharmaceutical industry) impacts upon trust or distrust in other systems (childhood vaccination). Also drawing on Giddens, Wong (2017) explores the relations of trust which underpin assisted reproduction and in particular, trust invested in the racialized identities of donors chosen through online profiles and how parental choices in the present will shape the future of their children to be. Trust, in this view, is linked with the expanding and differentiating markets of reflexive, reproductive choices, which are increasingly mediated and conducted “at a distance.”

Edwell and Jack (2017) consider how trust in biomedical experts and knowledge is splintered for pregnant women when they encounter the current controversies which characterize the diagnosis of gestational diabetes. Edwell and Jack maintain that lack of scientific consensus around the diagnosis and management of this condition indefinitely defers the conflict resolution that characterizes medical restitution narratives, i.e. in Arthur Frank’s (1994, 2013) typology of narratives aimed at the restitution of health. The absence of a clear finality in medical recommendations negatively impacts on the trust at the core of the provider–patient relationship, and pregnant women often embark on an online narrative quest for lay ways of dealing with such uncertainties and ontological troubles. In this way, expert knowledge in crisis, trust, subjectivity, and narrative are brought into close alignment.

Bowman’s (2017) essay offers a model for a fruitful dialogue between these diverse perspectives on scientific controversies, based on her own involvement as a bioethicist in non-hierarchical public engagement activities. Within a medical humanistic framework, Bowman collaborated in the production of creative re-enactments and inclusive discussions of bioethical case studies—in the form of radio programmes and theatre plays—which openly acknowledged the different levels of investment and expertise of all participants involved. Bowman concludes that a meaningful participation of the public in dissent, debate, and an ethics of uncertainty engenders and nurtures trust, as well as leads to innovative forms of effective public engagement.

## Narrating Trust

As noted, Edwell and Jack (2017) perhaps most obviously underline the centrality of narrative for trust’s ontological dimensions. In response to a faltering medical plotline that has been imposed on them, women often initiate a narrative quest as a method for navigating their pregnancy in light of the undecided, contradictory biomedical approaches to gestational diabetes. However, narrative is also implied in the relations of trust with which all our contributors contend and which is implied by the idea of experts and publics. Narratives are not simply written and recorded: they are read and heard (Andrews, Tamboukou, and Squire 2013; Squire et al., 2014). This telling and listening relation of narrative is centred on the communicative action which constructs public trust in expert knowledge systems of contemporary biomedicine. Moreover, who gets to tell their story and when and who is prepared to listen and take note is also implied in narratives on trust (Davis and Manderson 2014; Plummer 1995). To different extents, all of our contributors deal with the narrative implications of the relationships through which trust acquires its potency as a social form.

Narrative’s nuance and complexity, its affective richness, and its ability to move both teller and listener as it is told are implicated in the practice of bioethics for Bowman (2017). The narrative power of experts is profitably challenged in the play and radio programme she describes, which advocate an empowering multiplicity of narrators and sources of knowledge to reconceptualize public trust in experts.

Blending rhetoric and narrative medicine, Edwell and Jack (2017) build upon Arthur Frank’s categorization of illness narratives and postulate that medical uncertainty unsettles the conventional plotline of rewarding quest that underpins stories of medical triumph. More interestingly for this symposium, when healthcare professionals voice the need for further research in their interactions with pregnant women, without acknowledging the existence of a controversy regarding diagnosis and management which can be easily discovered through a simple google search, not only do they defer a symbolic resolution of a narrative query, they also defer the possibility of being perceived as sources of trust and knowledge. At the same time, when diagnosed with gestational diabetes, otherwise conscientious pregnant women are implicitly labelled as untrustworthy around their alleged lifestyle choices. Thus, by contrasting expert and public accounts of gestational diabetes, Edwell and Jack ultimately shed light on the narrative construction of biomedical knowledge and controversy.

The article by Wong (2017), while not ostensibly engaged with narrators and audiences, nevertheless indicates that the online profiles of donors are read by recipients to help them choose what they assume to be the characteristics of their child, in a process aimed at securing the trust of that future child. Donors in this sense depict themselves according to racialized and other social norms which figure in assisted reproduction. Recipients are, therefore, also bound into narrative interpretation, both of the social characteristics of donors and their own biographical futures as parents. This commercial relationship, conducted through the faceless commitments of donors and recipients, exhibits itself then as both one of trust and one articulated through the narrator-reader relation.

Atwell et al. (2017) provide insight into how vaccine refusers tell their stories of scepticism and resistance and how they position themselves in relation to both the commercialism of Big Pharma and public health imperatives on health and childhood immunization. These insights guard against overly simplistic characterization of vaccine refusal and the related tendency to oppose expert knowledge with lay perspectives and therefore to blame those who appear to be resistant. The paper thus poses important questions for example with how bioethics and policymaking have, for the most part, dealt with refusals to vaccinate on grounds of irrationality.

Without actually presenting narrative fragments, Buchman et al. (2017) nevertheless consider narratives on the experience of chronic pain with particular reference to what they typify as the epistemic injustice which accrues in the biomedical management of longstanding, difficult to treat pain. As Bowman (2017) contends, the fault lines in the public–expert relation across the fields of chronic pain, childhood vaccination, commercialized assisted reproduction, and gestational diabetes, for example, open up possibilities for alternative ways of articulating narrative relations on expert knowledge and trust.

## Expert Knowledge and Engagement

The contributions to this symposium also address, in different ways, the importance of expert knowledge systems for individual and collective life projects. But expert knowledge, particularly that which arises from science, has a probabilistic, uncertain character. Trust becomes important, therefore, in circumstances when the future cannot be known with certainty (Barbalet 2009). Trust, in this sense, acquires performativity which allows individuals to move into the future without certain knowledge. This feature of trust—its relation with not knowing—is perhaps its most troublesome effect in efforts to engage publics with expert knowledge. Partial knowledge, provisional assessments of the likelihood of future events, and continual revision of presiding assumptions are knowledge practices axiomatic to scientific inquiry but less easy to communicate to the public—particularly those pitched into crises around their health or making intensely technical and scientific decisions about their futures and that of their progeny.

This mismatch between forms of knowledge is linked to the problematic imposition of expertise and the challenge to hierarchies of knowledge on the part of publics. As Buchman et al. (2017) show with regard to chronic pain, the epistemically-privileged status of biomedical experts in therapeutic relationships bestows on them additional responsibility to take into account patient narratives on pain, its management, and their responses to therapeutic interventions, in order to rectify epistemic asymmetries. Pain, through its distressing subjectivity, is perhaps one of the keenest instances of the biomedical realm where personal experience comes closest to erasure in biomedical epistemology. For Buchman et al. these paternalistic epistemologies need to be questioned and displaced with “epistemic humility” to avoid the risk of epistemic injustice, originally defined by Fricker as “a type of harm that is done to individuals or groups regarding their ability to contribute to and benefit from knowledge” (Fricker 2007). In this regard, Buchman et al.’s paper connects with Bowman’s call for uncertainty and provisionality as the basis for more creative engagements with knowledge and the trust relations on which it depends.

For Attwell et al. (2017) and their research on vaccine refusal, disentangling trust in individuals from trust in systems is not always possible because of the inherently relational, and mediated, character of trust. As they note, Giddens referred to the “meeting places” of individuals and institutions as “access points,” or, in other terms, points of articulation of publics and expert knowledge systems. Implied therefore is the idea that the access points across biomedicine—the doctor–patient relationships, genetic counselling, assisted reproduction websites, patient consent forms, and so on—are important sites for public engagement. This more systemic view of public engagement with biomedical knowledge helps to decentre what is likely to be undue focus on what individuals and publics do not know and do not do and, as in the case of vaccine refusers, their resistances. In this view, the promotion of, for example, childhood vaccination can refocus on the range of ways in which publics are invited into engagement with vaccine technologies. In the case of vaccination, it also becomes of paramount importance not to reject parents’ decisions to refuse vaccination on the basis of a supposed “deficit of knowledge.” Instead, it becomes important to recognize that there are other values in their narratives that can explain their refusal to vaccinate (and that need to be taken into consideration for any meaningful policy response).

Echoing Attwell et al., Wong’s (2017) paper indicates that gamete agency websites are portrayed and function as “access points of abstract expert systems” (Giddens 2013a quoted in Wong 2017, ¶1). The websites are portals for public engagement with the knowledge systems of assisted reproduction. But as Wong makes explicit, service users invest in their futures on the basis of trust. For those using egg donation websites, trust is placed not only in the mediated service itself but also in the idea of “the future child”—somebody who does not yet exist but whose identity is brought into existence by trust in the online exchanges. Wong shows, too, how the communication of trust is increasingly influenced by our high choice media environments. The online platforms, suggests Wong, “facilitate gamete acquisition” by relying on “being able to trade in trust.” This mediation of trust lends it commodity value, in terms of the marketing of the service to users and in the idea that consumers avail themselves of trust through their choices. These commercial providers therefore market trust, engaging publics in making choices for their futures. Given the rise of consumer oriented healthcare and the growing range of self-diagnostic technologies (Lupton 2015), Wong’s account of assisted reproduction serves as a harbinger of the future organization of public engagements with biomedical knowledge and its technologies.

Edwell and Jack (2017) make the point that when individuals’ experiences and values are not incorporated into the knowledge systems of biomedicine, the epistemic gap between individuals and medical experts can lead to “risk-controversies.” Edwell and Jack (2017) problematize these ruptures of trust at different levels: pregnant women lose trust in their ability to make healthy lifestyle choices and healthcare professionals label them as “untrustworthy,” while scientific dissensus undermines trust in biomedicine. The way ahead, the authors suggest, is to involve publics in scientific debate more actively.

Bowman’s paper rearticulates the notion of what counts as public engagement work, and how it can be conceptualized. The paper argues that public engagement with questions of ethics (and science and biomedicine) might inadvertently replicate the hierarchical and patronizing structure of much biomedical knowledge exchange. To avoid this pitfall, the epistemic asymmetries between experts and the public need to be subverted, and the contribution and meaningful participation of lay voices needs to be sought from the beginning of process of public engagement.

## Reflections

This symposium argues that trust is not necessarily positioned antithetical to distrust and that we need to move away from hydraulic and binary notions of trust to articulate its complexities in expert knowledge systems, which are necessarily relational and mediated. This view gives emphasis to the narrative implications of the relationships through which trust acquires its performativity as a way to navigate uncertain futures.

Engagement with expert knowledge presupposes the creation of alternative types of narratives, outside the usual ones presented by scientists that assume a deficit of scientific knowledge on the part of individuals. Trust as the basis of biomedicine and healthcare is not necessarily always benign: an uncritical approach to trust may become disciplinary and may reinforce epistemic asymmetries inherent in medicine and healthcare. A new kind of attention is needed to avoid the reinscription of epistemic asymmetries in public engagement forms.

Significant questions are also raised about the nature of expertise, and who can be regarded as an expert. It does seem to be the case that we are witnessing a backlash against experts in different domains—including in bioethics itself, even though the discipline was created to assuage mistrust in medical care (Ashcroft 2004; Wilson 2014). The contributions to this symposium indicate that epistemic chasms, knowledge hierarchies, and a deferral and avoidance of uncertainty, may contribute to the crisis of expertise. A deeper analysis and articulation of why this may be the case is needed.

Finally, the uncritical quest to simply further and secure trust may also be part of the problem. As we noted, shifting the gaze away from questions of whether or not publics trust experts, science, and knowledge (i.e. from hydraulic and binaries notions of trust) towards the ethics, social relations, and meanings of trustworthiness, opens up critical space for analysis of the qualities and conduct of experts, the practices of science, including its power hierarchies, and onto knowledge itself.

We hope with this symposium to have opened up the floor for many more questions, and look forward to seeing how these may be taken up.

## References

[CR1] Andrews M, Tamboukou M, Squire C (2013). Doing narrative research.

[CR2] Ashcroft, R.E. 2004. Bioethics and conflicts of interest. *Studies in History and Philosophy of Science Part C: Studies in History and Philosophy of Biological and Biomedical Sciences* 35(1): 155–165.

[CR3] Attwell, K., J. Leask, S.B. Meyer, P. Rokkas, and P. Ward. 2017. Vaccine rejecting parents’ engagement with expert systems that inform vaccination programs. *Journal of Bioethical Inquiry* 14(1): doi. 10.1007/s11673-016-9756-7. 10.1007/s11673-016-9756-7 27909947

[CR4] Barbalet J (2009). A characterization of trust, and its consequences. Theory and Society.

[CR5] Beecher, H.K. 1976. Ethics and clinical research. In *Biomedical ethics and the law*, edited by J.M. Humber and R.F. Almeder, 193–205. Springer US.

[CR6] Bishop JP (2008). Rejecting medical humanism: Medical humanities and the metaphysics of medicine. Journal of Medical Humanities.

[CR7] Bowman, D. 2017. The moral of the tale: Stories, trust, and public engagement with clinical ethics via radio and theatre. *Journal of Bioethical Inquiry* 14(1): doi. 10.1007/s11673-016-9766-5. 10.1007/s11673-016-9766-5 PMC5340826 28063105

[CR8] Briggs C, Hallin D (2016). Making health public: How new coverage is remaking media, medicine, and contemporary life.

[CR9] Brooks M (2016). Mosquitoes and Zika: Time to harness genetic modification?. British Medical Journal.

[CR10] Buchman, D.Z., A. Ho, and D.S. Goldberg. 2017. Investigating trust, expertise, and epistemic injustice in chronic pain. *Journal of Bioethical Inquiry* 14(1): doi. 10.1007/s11673-016-9761-x. 10.1007/s11673-016-9761-x 28005251

[CR11] Calnan M, Rowe R (2008). Trust matters in healthcare.

[CR12] Charon R (2001). Narrative medicine: A model for empathy, reflection, profession, and trust. JAMA.

[CR13] Davis M, Manderson L (2014). Disclosure in health and illness.

[CR14] Doudna J, Charpentier E (2014). The new frontier of genome engineering with CRISPR-Cas9. Science.

[CR15] Edwell, J., and J. Jack. 2017. Gestational diabetes testing, narrative, and medical distrust. *Journal of Bioethical Inquiry* 14(1): doi. 10.1007/s11673-016-9762-9. 10.1007/s11673-016-9762-9 28005250

[CR16] Frank, A.W. 2013, 1994. *The wounded storyteller: Body, illness, and ethics*. University of Chicago Press.

[CR17] Freimuth V, Musa D, Hilyard K, Quinn S, Kim K (2013). Trust during the early stages of the 2009 H1N1 Pandemic. Journal of Health Communication.

[CR18] Fricker, M. 2007. *Epistemic injustice: Power and the ethics of knowing*. Oxford University Press.

[CR19] Fukuyama F (1995). Trust: The social virtues and the creation of prosperity.

[CR20] Giddens A (1990). The consequences of modernity.

[CR21] ———. 1991. *Modernity and self identity: Self and society in the late modern age*. London: Polity.

[CR22] Hardin R (2006). Trust.

[CR23] Kass NE, Sugarman J, Faden R (1996). Trust the fragile foundation of contemporary biomedical research. Hastings Center Report.

[CR24] Lupton D (2015). Health promotion in the digital era: A critical commentary. Health Promotion International.

[CR25] Luxon N (2015). Foucault on freedom and trust. The Review of Politics.

[CR26] O’Neill O (2002). A question of trust: The BBC Reith Lectures 2002.

[CR27] Pellegrino ED (1995). Toward a virtue-based normative ethics for the health professions. Kennedy Institute of Ethics Journal.

[CR28] ———. 1985. The virtuous physician, and the ethics of medicine. In *Virtue and medicine*, edited by E.E. Shelp, 237–255. Springer Netherlands.

[CR29] Plummer K (1995). Telling sexual stories: Power, change and social worlds.

[CR30] Putnam R (2000). Bowling alone: The collapse and revival of American community.

[CR31] Sherrieb K, Norris F, Galea S (2010). Measuring capacities for community resilience. Social Indicators Research.

[CR32] Squire C, Davis M, Esin C (2014). What is narrative research?.

[CR33] Wilson, D. 2014. *The making of British bioethics*. Manchester University Press. 25320834

[CR34] Wong, K-A. 2017. Donor conception and “passing,” or; Why Australian parents of donor-conceived children want donors who look like them. *Journal of Bioethical Inquiry* 14(1): doi. 10.1007/s11673-016-9755-8. 10.1007/s11673-016-9755-8 28108866

